# Cochlea sparing optimized radiotherapy for nasopharyngeal carcinoma

**DOI:** 10.1186/s13014-021-01796-4

**Published:** 2021-04-01

**Authors:** Enkelejda Lamaj, Erwin Vu, Janita E. van Timmeren, Chiara Leonardi, Louise Marc, Izabela Pytko, Matthias Guckenberger, Panagiotis Balermpas

**Affiliations:** Department of Radiation Oncology, University Hospital Zurich (USZ), University of Zurich (UZH), Rämistrasse 100, 8091 Zurich, Switzerland

**Keywords:** Nasopharyngeal carcinoma, Cochlea sparing, Late toxicity, Chemoradiotherapy, VMAT

## Abstract

**Background:**

Definitive chemoradiotherapy (CRT) is standard of care for nasopharyngeal carcinoma. Due to the tumor localization and concomitant platinum-based chemotherapy, hearing impairment is a frequent complication, without defined dose-threshold. In this study, we aimed to achieve the maximum possible cochleae sparing.

**Materials and methods:**

Treatment plans of 20 patients, treated with CRT (6 IMRT and 14 VMAT) based on the QUANTEC organs-at-risk constraints were investigated. The cochleae were re-delineated independently by two radiation oncologists, whereas target volumes and other organs at risk (OARs) were not changed. The initial plans, aiming to a mean cochlea dose < 45 Gy, were re-optimized with VMAT, using 2–2.5 arcs without compromising the dose coverage of the target volume. Mean cochlea dose, PTV coverage, Homogeneity Index, Conformity Index and dose to other OAR were compared to the reference plans. Wilcoxon signed-rank test was used to evaluate differences, a *p *value < 0.05 was considered significant.

**Results:**

The re-optimized plans achieved a statistically significant lower dose for both cochleae (median dose for left and right 14.97 Gy and 18.47 Gy vs. 24.09 Gy and 26.05 Gy respectively, *p* < 0.001) compared to the reference plans, without compromising other plan quality parameters. The median NTCP for tinnitus of the most exposed sites was 11.3% (range 3.52–91.1%) for the original plans, compared to 4.60% (range 1.46–90.1%) for the re-optimized plans (*p* < 0.001). For hearing loss, the median NTCP of the most exposed sites could be improved from 0.03% (range 0–99.0%) to 0.00% (range 0–98.5%, *p* < 0.001).

**Conclusions:**

A significantly improved cochlea sparing beyond current QUANTEC constraints is feasible without compromising the PTV dose coverage in nasopharyngeal carcinoma patients treated with VMAT. As there appears to be no threshold for hearing toxicity after CRT, this should be considered for future treatment planning.

**Supplementary Information:**

The online version contains supplementary material available at 10.1186/s13014-021-01796-4.

## Background

Definitive chemoradiotherapy (CRT) with or without (neo)adjuvant chemotherapy is the standard treatment for nasopharyngeal carcinomas (NPC). With increasing improvements of local control rate and overall survival [1], the management of late toxicities becomes more important [2]. Due to the tumor location, hearing impairment is a common and well-described complication after treatment [3]. Although this toxicity mostly depends on the radiation dose to the cochlea, only limited data for the relation between radiation dose and hearing impairment is available. Several studies have tried to find a threshold of mean or median cochlear dose (MCD) associated with hearing loss [4]. However, the small volume and consequently the difficult delineation as well as the different clinical situations and heterogeneity of cases and treatments hamper these analyses. Prospective data suggested that hearing loss was associated with the total dose received by the inner ear [5]. Moreover, an increased probability for hearing loss after combination with ototoxic chemotherapy, especially with cisplatin, has been often demonstrated before [6].

While a dose-volume analysis of the cochlea is not widely used, due to the small volume of the organ, the available clinical data suggest a strict limitation of radiation dose to the cochlea [7, 8], although there appears to be no clear threshold for avoiding late sequela. Furthermore, the majority of the data, including the QUANTEC recommendations originate from an era, where 3D and even 2D planning was common and a clear and unambiguous “safe” dose to the cochlea, especially for the specific case of patients with NPC has not been defined yet.

With this planning study we pursue the goal of exploiting the best possible cochlea sparing using most modern volumetric-modulated arc therapy (VMAT)-planning. The main hypothesis is that via most modern techniques, like state-of-the-art VMAT planning, improved cochlea sparing is possible without compromising plan quality. To achieve this goal without compromising the dose coverage of the target volume (PTV), or the sparing of other important organs at risk (OARs), we identified 20 patients treated in our cancer center, recontoured the cochleae and re-optimized treatment plans focusing on the maximum possible protection of the cochleae without compromising any other parameter. The optimized plans were finally compared to the original treatment plans in several statistical analyses.

## Patients and methods

Radiotherapy plans of twenty patients diagnosed with nasopharyngeal carcinoma between 2011 and 2019 were included in this study. Seventeen patients underwent curative chemoradiotherapy (CRT) while three patients only received radiotherapy (RT). Contouring of clinical target volumes was performed according to international guidelines [9] and previous RTOG recommendations for older cases [10]. All patients treated with concomitant CRT received platinum-based treatment. Details on the patient's characteristics are shown in Table 1. Every patient consented to anonymous data collection and the retrospective evaluation of patient and treatment data was approved by the local ethics committee (ProjectID 2019-00684).

**Table 1:** Patient characteristics

Characteristic	Value
Total (n)	20
Average age (years)	51.5 (range 24–82)
Gender (n)	
Male	16
Female	4
Mean cochlea volume (cm^3^)	
Left cochlea	0.215 (range 0.09–0.54)
Right cochlea	0.221 (range 0.08–0.45)
Mean prescription dose (Gy)	69.78 (range 66–70)
Concurrent chemotherapy (n)	
Cisplatin	12
Carboplatin (+ taxol/5-FU)	5
None	3
Performance status before RT start (n)	
ECOG 0	12
ECOG 1	6
ECOG 2	2
T status (n)	
T1	4
T2	8
T3	2
T4	6
N status (n)	
N0	10
N1	3
N2	5
N3	2
M status (n)	
M0	19
M1	1

Patients had been treated with volumetric-modulated arc therapy (VMAT) (n = 14) or step and shoot intensity modulated radiotherapy (IMRT) technique (n = 6) and 6 MV photons with a mean delivered dose of 69.78 Gy (range 66–70 Gy) and single doses of 2.0–2.12 Gy. Planning CT was acquired with a 2 mm slice thickness and individual thermoplastic masks were used for immobilization. Two experienced radiation oncologists independently recontoured the cochleae for every case according to an international consensus guideline [11] and reviewed the cases afterwards. While no relevant differences have been found, a consensus has been made for slightly varying contours. Target volumes and all other organs at risk remained unchanged. These cochlea-structures were expanded with a 3 mm margin as a PRV approach. The initially treated plans were then optimized in Eclipse Treatment Planning System (TPS) (Varian, San Diego, USA) with the aim of sparing the cochlea as much as possible, without compromising the PTV coverage or any other plan-quality parameter. For each patient a new VMAT plan was created for 6 MV and a maximum dose rate of 600 MU/min with a 178 number of segments for a full arc calculated in every 2 degrees gantry angle.

**Fig. 1:**
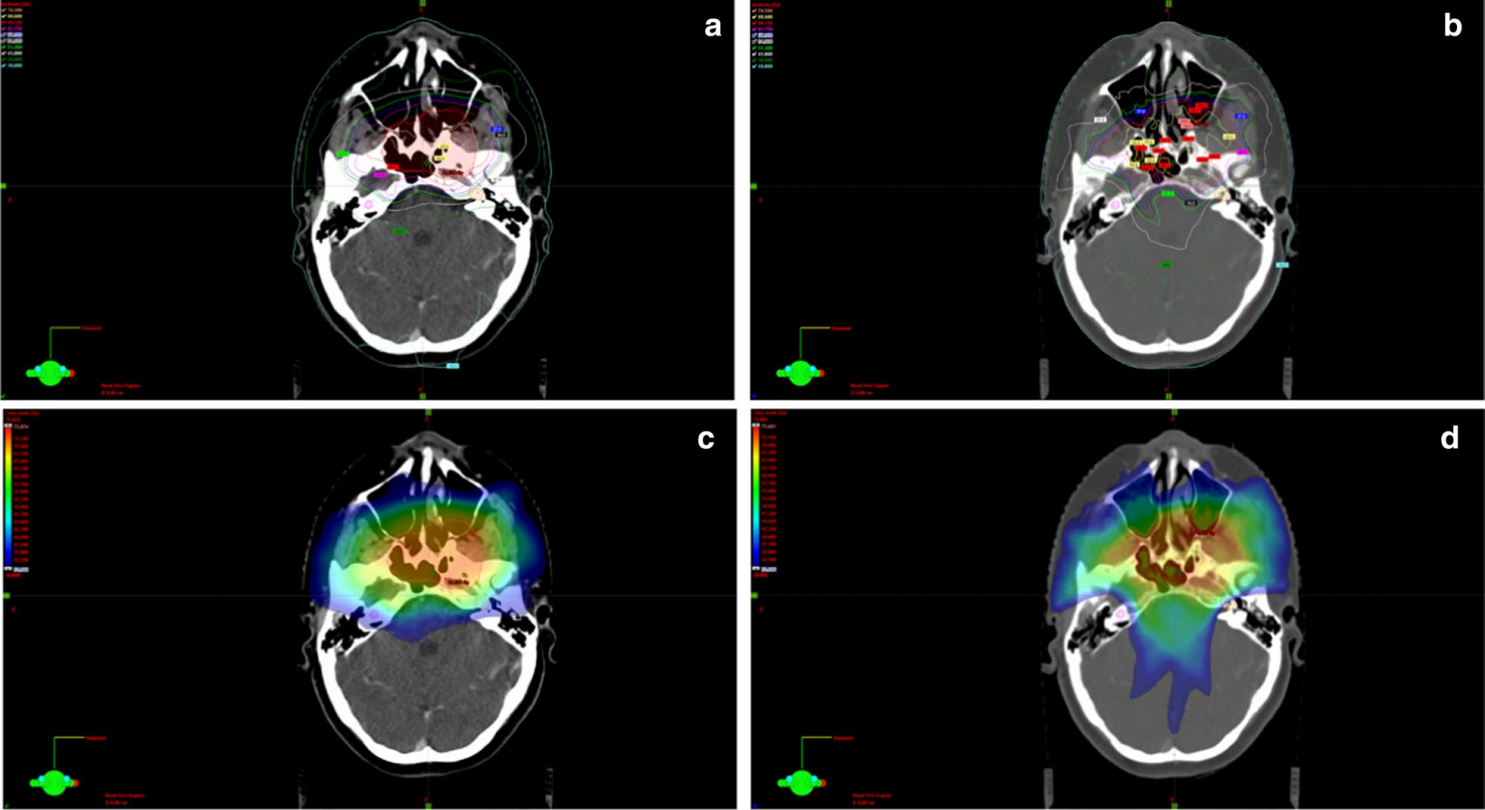
Exemplary comparison between original (**a**, **c**) and re-optimized plan (**b**, **d**)

Two or three coplanar or non-coplanar arcs were used according to the complexity and location of the PTV. The field size was defined by the jaw openings allowing for a jaw tracking in the optimizer. For the counterclockwise arcs (CCW) the jaw was closed to 2 cm from the isocenter on the × 1 axis and on the × 2 axis for the clockwise arcs (CW), allowing for better cochlear sparing. The collimator angle for each plan was individually chosen from minimum ± 5 degrees based on the patient anatomy. A rapid arc model (RAM) was used for the optimization. Objectives and priorities for the OARs and/or target volumes were changed when needed so that an optimal plan could be achieved. The main differences in the re-planning were the cochlea optimization-structures used in the optimizer and the optimization way. We expanded the cochlea with 3 mm margin to ensure sparing of the original structure. In the optimizer, both expanded and original structures were used. Furthermore, we used a Rapid arc model in the optimizer and “manual” normal tissue objectives for all plans (this was the case only in some of the original plans). Also, different collimator angles and jaw openings were implemented. More precise, for all cases of the re-optimized plans following procedures were applied: the jaws were closed for each beam so that the cochlea would remain outside the treatment field when possible, or as much as close to the cochlea if very close to the PTV. On the CW arcs the right cochlea was outside the treatment field, while the left one on the CCW arcs. Jaw tracking on the optimizer was allowed. Regarding the collimator angles the differences were minimal: 5-degree and 355-degree angles were often used for the re-optimized plans, while for the reference plans 3 degrees and 357 were more common. In the few cases of overlapping between the cochlea and the PTV we aimed to spare the cochlea on the opposite side, similar to the original plan. The resulting plan was re-optimized until a clinical acceptable plan was achieved. An example for the comparison between original and re-optimized plan is shown in Fig. 1.

Several planning variables, such as mean cochlea dose, PTV coverage, Dmean, Dmax, D98%, V95%, D2%, Homogeneity Index (HI) (defined as (D2%–D98%)/Dprescribed), RTOG’s Conformity Index (CI: Conformity Index = VRI/TV) [12] and dose to other OARs were compared to the reference plans. Several groups previously published normal tissue complication probability (NTCP) models for both tinnitus [13] and hearing loss [14, 15]. The fitted parameters of the Lyman-Kutcher-Burman (LKB) model were extracted from these studies and used to calculate the NTCP for both original and re-optimized plans. For tinnitus, TD_50_ = 46.52 Gy and m = 0.35 (Lee et al. 2015) were applied, whereas for hearing loss, TD_50_ = 51.7 Gy and m = 0.14 (Cheraghi et al. 2017). The following formula was utilized to perform NTCP modeling (the volume effect parameter n was set to 1 and MD was defined as the mean dose to the cochlea receiving the greater dose):

$$NTCP = \frac{1}{2\pi }\mathop \smallint \limits_{ - \infty }^{t} \exp \left( {\frac{{ - x^{2} }}{2}} \right)dx\quad {\text{with}}\quad t = \frac{{MD - TD_{50} }}{{m \cdot TD_{50} }}$$

Wilcoxon signed-rank test was used to evaluate differences in the cochleae sparing, and Wilcoxon rank-sum test was used to evaluate the relationship between tumor stage and cochleae doses where a *p* value below 0.05 was considered significant. All plans were in accordance with the International Commission on Radiation Units (ICRU 83) report [16].

## Results

**Fig. 2:**
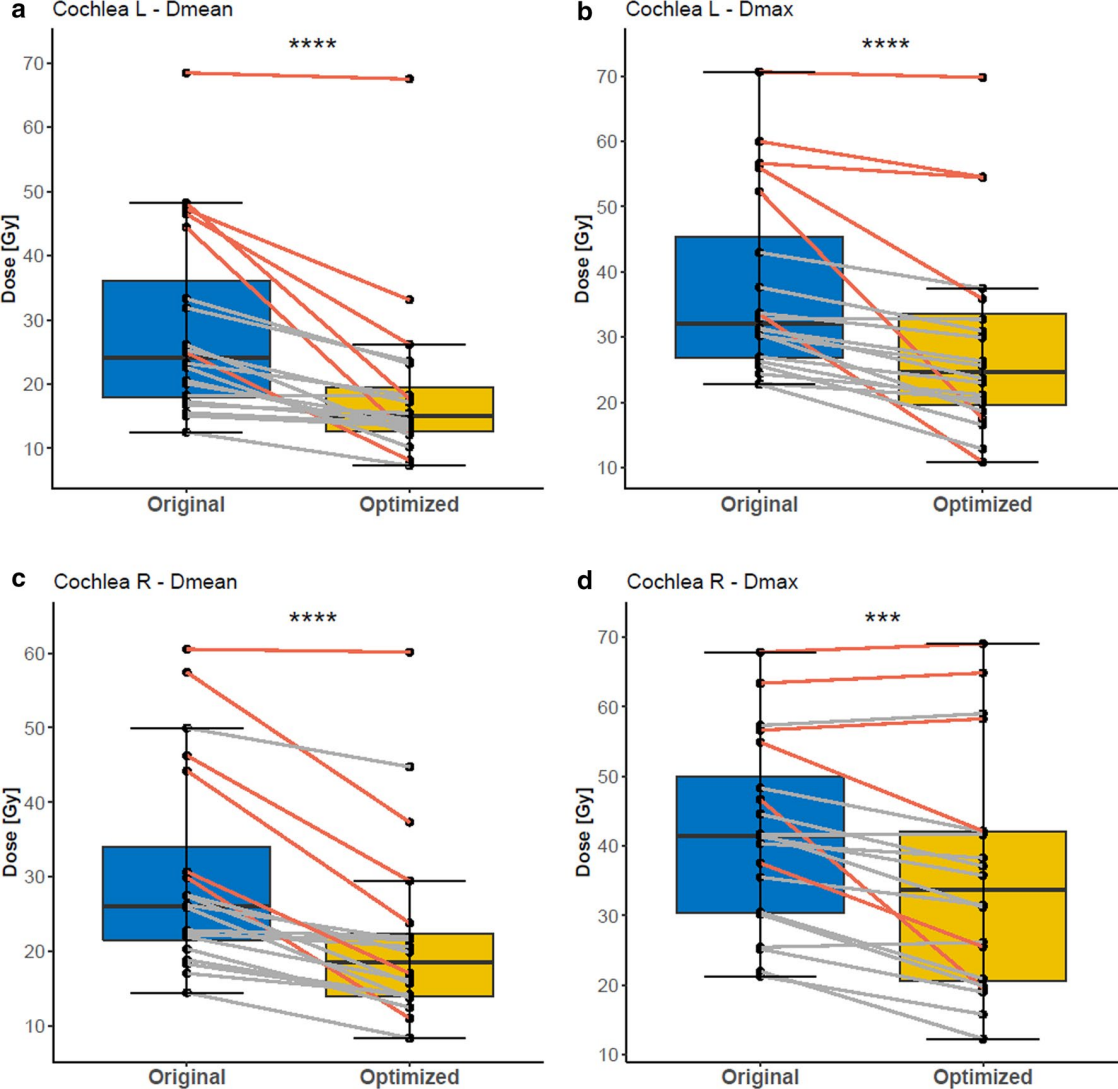
Boxplots for the cochlear mean and max doses of the original and optimized plans. Red lines indicate the patients that were originally planned with IMRT (n = 6). Stars indicate significance. ***p ≤ 0.001, ****p ≤ 0.0001

The mean volume of all cochlea contours was 0,218 ml (median 0.195 ml), comparable to the literature (0.13–0.56 ml) [5, 17]. Compared to the original treatment plans, cochlea sparing re-optimization resulted in a significant decrease of radiation dose for both cochleae (Table 2). For the left cochlea median Dmean was reduced from 24.09 Gy (range 12.52–68.45 Gy) to 14.97 Gy (range 7.31–67.48 Gy, *p* < 0.001) (Fig. 2). This was achieved for the right cochlea as well, where the median Dmean of 26.05 Gy (range 14.46–60.53 Gy) for the original plan was improved to a median Dmean value of 18.47 Gy (range 8.37–60.14 Gy, *p* < 0.001). As shown in Fig. 2, also median Dmax for the left cochlea (from 32.12 to 24.64 Gy, *p* < 0.001) and median Dmax for the right cochlea (from 41.47 to 33.66 Gy, *p* = 0.001) were significantly reduced. In the optimized plans it was possible to achieve bilateral cochleae doses < 45 Gy for 18/20 patients. The evaluation of influence of T-, N- and UICC^8th^-stage on the cochleae doses is presented in Table 3. Comparing the different stages, Dmean of the right cochlea is significantly higher for T3 and T4 tumors compared to T1 and T2 (*p* = 0.03).

**Table 2:** Comparison of dosimetric values of the original and re-optimized plans

Organ and dosimetric parameter	Original plans (median value)	Optimized plans (median value)	*p* value
Left cochlea Dmean	24.09 Gy (range 12.52–68.45 Gy)	14.97 Gy (range 7.31–67.48 Gy)	< 0.001
Right Cochlea Dmean	26.05 Gy (range 14.46–60.53 Gy)	18.47 Gy (range 8.37–60.14 Gy)	< 0.001
Left Cochlea Dmax	32.12 Gy (range 22.75–70.66 Gy)	24.64 Gy (range 10.86–69.8 Gy)	< 0.001
Right cochlea Dmax	41.47 Gy (range 21.29–67.87 Gy)	33.66 Gy (range 12.28–69.05 Gy)	0.001
PTV Dmean	69.96 Gy (range 66.00–70.42 Gy)	69.96 Gy (range 66.00–71.23 Gy)	0.201
PTV HI	0.095 (range 0.05–0.14)	0.070 (range 0.04–0.13)	0.001
PTV CI	0.595 (range 0.51–0.77)	0.595 (range 0.54–0.79)	0.046
PTV D2%	72.47 Gy (range 69.35–73.65 Gy)	71.98 Gy (range 67.86–73.54 Gy)	0.001
PTV D98%	65.99 Gy (range 62.91–68.75 Gy)	66.85 Gy (range 62.94–68.97 Gy)	< 0.001
PTV V95%	97.49% (range 92.73–99.8%)	98.73% (range 94.3–100%)	0.001
Brainstem Dmax	50.59 Gy (range 20.23–62.57 Gy)	50.81 Gy (range 20.38–64.96 Gy)	0.965
Left parotis Dmean	24.95 Gy (range 14.49–62.21 Gy)	23.68 Gy (range 14.53–61.61 Gy)	0.024
Right parotis Dmean	24.59 Gy (range 16.94–50.4 Gy)	21.92 Gy (range 15.43–50.4 Gy)	0.009
Spinal cord Dmax	40.27 Gy (range 33.69–54.01 Gy)	38.99 Gy (range 33.45–54.04 Gy)	0.011
Oral cavity Dmean	30.41 Gy (range 22.74–44.03 Gy)	31.56 Gy (range 23.16–42.41 Gy)	0.452
Mandible Dmean	30.03 Gy (range 21.16–37.37 Gy)	30.46 Gy (range 20.97–36.96 Gy)	0.985
Mandible V70Gy	0.000 (range 0–0.258)	0.000 (range 0–0.246)	0.789
Chiasm Dmax	10.70 Gy (range 3.12–60.23 Gy)	9.21 Gy (range 3.14–60.23 Gy)	0.231
Chiasm D0.03 cc	8.68 cc (range 2.99–57.17 cc)	7.33 cc (range 3.03–67.67 cc)	0.956
Left optic nerve D0.03 cc	12.94 cc (range 2.51–70.16 cc)	9.58 cc (range 2.48–69.81)	0.076
Right optic nerve D0.03 cc	17.35 cc (range 2.86–58.31 cc)	12.71 cc (range 2.86–57.06)	0.007
NTCP tinnitus	11.33% (range 3.52–91.10%)	4.60% (range 1.46–90.10%)	< 0.001
NTCP hearing loss	0.03% (range 0.00–98.97%)	0.00% (range 0.00–98.54%)	< 0.001

**Table 3:** Cross table with the influence of T-, N- and UICC^8th^-stadium on the cochleae doses

	T-stage		*p* value
	T1 or T2 (n = 12)	T3 or T4 (n = 8)	
Left cochlea Dmean			
Mean ± SD	15.4 ± 7.1	24.2 ± 18.1	*0.10*
Median [range]	14.0 [7.3–33.1]	18.2 [12.1–67.5]	
Left cochlea Dmax			
Mean ± SD	25.8 ± 12.2	34.0 ± 18.6	*0.34*
Median [range]	23.3 [10.9–54.5]	28.6 [17.5–69.8]	
Right cochlea Dmean			
Mean ± SD	16.3 ± 4.6	30.3 ± 16.2	***0.03***
Median [range]	16.0 [8.4–23.8]	25.5 [14.0–60.1]	
Right cochlea Dmax			
Mean ± SD	28.9 ± 9.7	45.4 ± 20.6	*0.13*
Median [range]	28.7 [12.3–42.2]	50.0 [15.8–69.1]	

	N-stage		*p* value
	N0 or N1 (n = 13)	N2 or N3 (n = 7)	
Left cochlea Dmean			
Mean ± SD	21.1 ± 15.4	15.0 ± 5.8	*0.35*
Median [range]	17.2 [7.3–67.5]	13.5 [8.1–26.2]	
Left cochlea Dmax			
Mean ± SD	31.1 ± 16.0	25.4 ± 14.0	*0.39*
Median [range]	29.9 [12.9–69.8]	23.0 [10.9–54.5]	
Right cochlea Dmean			
Mean ± SD	23.4 ± 14.1	19.2 ± 9.6	*0.49*
Median [range]	17.1 [12.5–60.1]	20.7 [8.4–37.3]	
Right cochlea Dmax			
Mean ± SD	36.1 ± 16.1	34.5 ± 19.2	*0.88*
Median [range]	31.5 [15.8–64.9]	35.8 [12.3–69.1]	

	UICC^8th^		*p *value
	UICC I or UICC II (n = 8)	UICC III or UICC IV (n = 12)	
Left cochlea Dmean			
Mean ± SD	17.2 ± 8.0	20.1 ± 15.8	*0.97*
Median [range]	16.3 [7.3–33.1]	14.4 [8.1–67.5]	
Left cochlea Dmax			
Mean ± SD	29.3 ± 13.2	29.0 ± 17.0	*0.73*
Median [range]	26.8 [12.9–54.5]	24.3 [10.9–69.8]	
Right cochlea Dmean			
Mean ± SD	17.7 ± 3.9	24.7 ± 15.6	*0.73*
Median [range]	16.6 [12.5–23.8]	21.1 [8.4–60.1]	
Right cochlea Dmax			
Mean ± SD	31.5 ± 7.5	38.2 ± 20.7	*0.79*
Median [range]	31.4 [19.9–42.2]	38.7 [12.3–69.1]	

Significant values are written in bold

**Fig. 3:**
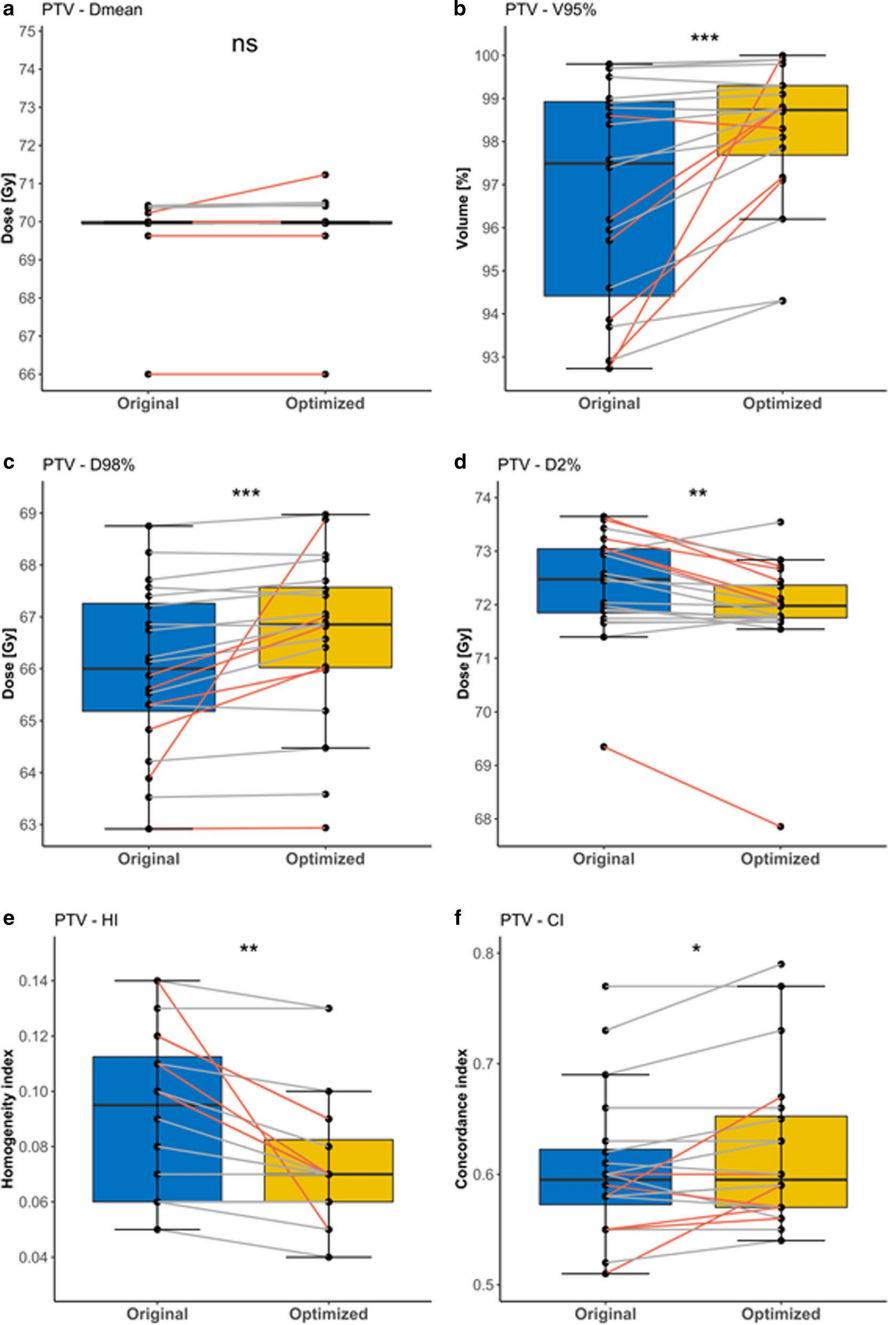
Boxplots for the PTV dose parameters of the original and optimized plans. Red lines indicate the patients that were originally planned with IMRT (n = 6). Stars indicate significance. *NS* non-significant, *p ≤ 0.05, **p ≤ 0.01, ***p ≤ 0.001

To evaluate the PTV coverage of the plans, we compared several parameters, which are shown in Fig. 3. For PTV-Dmean, no differences were observed between the original and re-optimized plans (69.96 Gy, *p* = 0.201), whereas all other PTV-related dose parameters significantly improved after re-optimization. Due to marginal differences in PTV D2% and PTV D98% in the old and new plans, the median PTV HI of 0.095 for the original plans improved to a median PTV HI of 0.070 (*p* = 0.001). Meanwhile, the median PTV CI of 0.595 did not change after re-optimization.

As the re-optimized plans required to be acceptable for clinical application, the other OARs had to meet the same constraints as in the original plans. A comparison of the most important OARs is presented in Table 2. While Dmax of the brainstem (median of 50.59–50.81 Gy, *p* = 0.965), Dmean of the mandible (median of 30.03 Gy and 30.46 Gy, *p* = 0.985) and Dmean of the oral cavity (median of 30.41 Gy and 31.56 Gy, *p* = 0.452) remained unchanged, the sparing of both parotids was improved by re-optimization from a median Dmean of 24.95–23.68 Gy (left parotid, *p* = 0.024) and 24.59–21.92 Gy (right parotid, *p* = 0.009). The same was observed for the spinal cord, where the median Dmax of 40.27 Gy was reduced to 38.99 Gy (*p* = 0.011). The boxplots for the OARs are shown in the Additional file 1.

The median number of Monitor Units (MU) amounted to 353.6 MUs for the CW and 386.1 MUs for the CCW compared to 286.9 MUs (CW) and 333.6 MUs (CCW) for the original plans (range 51.6–711.6 MUs and 192–663.8 MUs for the CW and CCW respectively). The range of MUs for the optimized plans resulted in 50.4 MU–723 MU for the CW arcs and 198.9 MU–716.3 MU for the CCW arcs.

The median NTCP for tinnitus of the most exposed sites was 11.3% (range 3.52–91.1%) for the original plans, compared to 4.60% (range 1.46–90.1%) for the re-optimized plans (*p* < 0.001). For hearing loss, the median NTCP of the most exposed sites could be improved from 0.03% (range 0–99.0%) to 0.00% (range 0–98.5%, *p* < 0.001).

## Discussion

Due to improvements in modern treatment of nasopharyngeal cancer patients, both in terms of local and systemic therapy [18, 19], the population of patients surviving this cancer diagnosis has been increasing in the last decades. This development leads to an increased relevance of controlling the late treatment-related toxicities with the goal of preserving quality of life. In this study, we were able to demonstrate that an optimized cochlea sparing intensity modulated radiotherapy planning is feasible without compromising PTV dose coverage in nasopharyngeal carcinoma patients, resulting potentially in improved hearing preservation.

Severe hearing impairment, one of the main late adverse effects of irradiation directed to the nasopharynx, is also influenced by several factors such as fractionation, age and chemotherapy and has been suggested to be proportional to the dose to the auditory apparatus. The cochlea appears to be the most radiosensitive part, with an α/β ratio of 2 [20]. Toxicities which might occur after irradiation of the cochlea include tinnitus and radiation-induced sensorineural hearing loss (SNHL). Significant SNHL is shown by serial audiological tests to occur in up to 50% of the nasopharynx patients receiving radiotherapy and is mainly explained by the irreversible destruction of the auditory sensory hair cells of the organ of Corti, especially outer hair cells of higher frequencies [21, 22]. Despite intense research [23], there still is a lack of data providing a clear dose threshold for cochlea toxicity. Over the years, several groups proposed different dose constraints for the cochlea. In 1991 Grau et al. [24] described a higher incidence of SNHL with an average of 21% for doses above 50 Gy. These results were endorsed by Pan et al. [5] in a case series which showed dose-dependent hearing loss for even lower cochlear radiation doses (45 Gy). Other groups also [8, 25, 26] supported these findings in retrospective analyses leading to the QUANTEC dose-volume constraints [27, 28] of cochlear mean doses < 45 Gy associated with an expected complication rate of < 30% if the treatment plan meets this constraint. This incidence of complications is however unacceptably high, especially if one considers the added toxicity of the most commonly used platinum-chemotherapy for these patients. An overview of current literature on radiation-induced ear toxicity is presented in Table 4.

**Table 4:** Overview of literature on radiation-induced ear toxicity

	Study design	Number of patients (n)	Concurrent chemotherapy	Mean cochlear dose	Radiation induced ear toxicity	
Pan et al. [5]	Prospective	31	No	47.4 Gy	Significant hearing loss for cochlear dose ≥ 45 Gy	Older patients more susceptible to hearing loss
Chen et al. (2006)	Retrospective	22	yes	48.5 Gy	57% SNHL	21% serous otitis
Van der Putten et al. [8]	Retrospective	52	No	36.1 Gy	32% hearing loss	39% tinnitus
Hitchcock et al. [39]	Prospective	62	34% RT only 66% yes	33.1 Gy	RT only group: SNHL for cochlear dose ≥ 40 Gy	Chemoradiation group: SNHL for cochlear dose ≥ 10 Gy
Chan et al. [26]	Retrospective	97	yes	48.9	9.4% low frequency SNHL	51.2% high frequency SNHL
Nutting et al. [30]	Prospective	110	No	56.2 Gy (3D-CRT) vs. 35.7 Gy (IMRT)	SNHL: 39% (3D-CRT) vs. 35% (IMRT)	Tinnitus: 58% (3D-CRT) vs. 44% (IMRT)
Bass et al. [31]	Retrospective	473	n.a	Exposure defined as cochlear dose > 1 Gy	22% Mild hearing impairment	38% severe hearing impairment

IMRT in various forms is considered worldwide as standard-of-care for treating nasopharyngeal cancer as it is associated with an increased OS compared to older techniques [29]. Following the broad implementation of this technique, the phase III trial COSTAR [30] demonstrated that IMRT for parotideal cancer could reduce the cochlea dose below the formerly accepted tolerance dose of 40–45 Gy to a mean of 35.7 Gy, but this did not lead to a significant reduction of SNHL at 12 months after RT. The authors therefore hypothesized that the previously accepted tolerance dose for cochlea irradiation is too high. This was supported by Lee et al. [13], who published a probability model for cochlea constraints which suggested a mean dose below 32 Gy to maintain the incidence of grade 2 + tinnitus toxicity under 20% and Wang et al. [6], who have observed a complete lack of SNHL only for patients receiving a median dose below 34 Gy to the cochlea. Moreover, in a recent trial examining long term survivors of childhood cancer treated a.o. with cochlea radiation with or without platin, the median doses of 26.6–28.2 Gy already caused clinically significant hearing impairment [31]. These considerations stress the urgent need for further dose reduction for this organ.

Similar to the studies above and based on the guidelines-recommendations, all of the historical clinically applied plans included in this planning study not only respected the QUANTEC-criteria, but with Dmean of 29–30 Gy provided mean cochlea doses even lower than previously achieved by Nutting et al. [30] or suggested by Lee et al. [13]. However, as this dose exposure seems to be still associated with considerable side effects for an important percentage of the patients, we aimed to explore the limits of cochlea sparing without jeopardizing PTV coverage or sparing of other important organs as parotids, mandible, optic pathway and spinal cord/brainstem. In this regard, the present analysis has achieved a significantly lower dose exposure of both cochleae, resulting in a Dmean < 20 Gy. Based on NTCP analyses, we were able to show that such an exposure would reduce the probability of both tinnitus and hearing loss significantly, resulting in a probability < 5% for tinnitus and practically negligible risk for hearing loss (at least if the contribution of cisplatin is not taken into account). Interestingly, we could also observe a general improvement of the plans, especially for patients originally treated with step-and-shoot IMRT, which reached statistical significance for some of the parameters like PTV-V95% and parotid sparing. These additional improvements are obviously a result of the more modern and sophisticated planning methods used here and although statistically significant are not clinically significant and beyond the scope of this manuscript. The optimized cochlea sparing was not achieved at the cost of worse target coverages like the maximal doses to brainstem or mandible or the mean dose to the oral cavity, which did not show any significant differences between original and re-optimized plans. Importantly, not a single quality parameter was inferior in the cochlea-optimized plans. Moreover, the median number of monitor units (MU) and the beam-on time of the cochlea-optimized plans were reasonable. These findings highlight the practicability of intensive cochlea sparing for daily routine.

There exist only very limited data regarding cochlea-sparing directed planning for nasopharyngeal carcinoma. In another planning trial implementing VMAT technique for head and neck cancer [32] even doses below 10 Gy were possible for unilateral cochlear sparing, but NPC patients were strictly excluded and the achieved unilateral sparing could also lead to other problems like tinnitus or vestibular defects. A similar study investigated a stratified cochlea-dose limitation IMRT-planning approach for nasopharyngeal cancer and reported Dmean of 43.8 Gy and 46.2 Gy for contralateral and ipsilateral cochlea respectively [33]. This dose exposure was significantly lower than in the non-optimized plans (where the dose to both sides amounted ca. 50 Gy), but still excessively higher compared to our data and international recommendations. Possible reasons for these differences could be the higher percentage of T3 tumors in the latter study (8/19 versus 2/20 in our cohort), although the number of T4 cases was identical (6), as well as the step-and-shoot IMRT-technique used for optimizing, whereas in our study only VMAT was implemented. The fact, that our data shows a significant increase of Dmean of the right cochlea in T3 and T4 tumors supports this hypothesis. Indeed, one of the few other planning studies introducing modern radiotherapy techniques for cochlea sparing could prove the superiority of VMAT to this regard when compared with step-and-shoot IMRT [34]. However, according to the current literature and the present results we think there is clearly more room for improvement for most cases, as a dose of 45 Gy could already be detrimental for hearing. This has been demonstrated a.o. in a prospective trial including various head and neck malignancies [5]. Such ambitious dose goals for cochlea-sparing could be feasible with modern, elaborate planning, as a small retrospective study demonstrated for 5 patients using tomotherapy [35]. Regarding tumors with clival involvement, as it was the case with the cT4 tumors presented here, the cochlea sparing becomes more challenging. As described in Table 3, the mean dose for right and left cochlea in the 6 cT4- and 2 cT3-cases amounted to 25.5 Gy and 18.2 Gy respectively, which is considerably (and in some cases significantly) higher than for smaller tumors, but still below the recommended 45 Gy. There were indeed some cases included with gross disease abutting the cochlea, as can be seen in the range of cochlea Dmean, with 67.5 Gy and 60.1 Gy being the highest exposures calculated for right and left cochlea respectively in this cohort. In similar cases, a maximum possible sparing of the contralateral side should be strictly recommended, which again stresses the importance of the findings of this study.

Recently, two large randomized trials have proven the superiority of additional induction chemotherapy for advanced nasopharyngeal carcinoma [36, 37] and a meta-analysis has previously confirmed this also for adjuvant chemotherapy [38]. Especially in this modern era of intensified systemic treatment, almost always including high-dose cisplatin-component, there is an urgent need for limiting radiation-related ototoxicity to the minimum. Interestingly, in a prospective trial of the University of Utah trying to evaluate the relative contribution of radiotherapy and cisplatin to hearing-loss of head and neck cancer patients, those receiving relatively low cisplatin-doses (unlike the actual recommendations for advanced nasopharyngeal carcinoma) experienced impairment already at 10 Gy exposure. In this trial, in order to avoid significant sequelae to the cochlea with radiotherapy alone, the dose should be below 40 Gy [39]. An analysis of medulloblastoma patients treated with modern radiotherapy techniques and concomitant chemotherapy showed that the risk of hearing loss below 35 Gy is practically negligible [3], which is in accordance with various NTCP-models [14, 15, 40] and with the clinical data of Lee [1] and Wang [6] reported above, so it is reasonable to pursue at least this, for most cases realistic goal, for cochlea sparing.

The present study has some limitations. First, all of the optimization has been only retrospectively conducted. Second, the exact and objective clinical value of this additional cochlear sparing remains unclear and a prospective trial, including both objective audiometric evaluation and patient reported outcomes could better investigate the added value of our findings. Finally, the original plans have not been developed with the same technique, using both IMRT and VMAT methods. Nevertheless, to the best of our knowledge, this is one of the first studies investigating the limits of cochlea sparing by means of VMAT-planning for a strictly homogeneous cohort of patients, all treated for nasopharyngeal carcinoma.

## Conclusions

Published data so far back the theory that the widely accepted QUANTEC constraint for the cochlear tolerance dose of 45 Gy is too high and there is no well-defined threshold for ototoxicity, especially for patients receiving chemotherapy. Our study was able to demonstrate that a substantially improved cochlea sparing is feasible without compromising the PTV dose coverage in the majority of patients without compromising sparing other organs at risk of compromising target coverage. The clinical effect of this optimization should be evaluated in future retrospective and prospective clinical trials.

## Supplementary Information

**Additional file 1: Figure S1**. Boxplots for the dose parameters of brainstem, oral cavity, spinal cord and parotid of the original and optimized plans. Red lines indicates the patients that were originally planned with IMRT (n = 6). Stars indicate significance. NS: non-significant, *: p ≤ 0.05, **: p ≤ 0.01. **Figure S2**: Boxplots for the dose parameters of eye, optic nerve and chiasm of the original and optimized plans.Red lines indicates the patients that were originally planned with IMRT (n = 6). Stars indicate significance. NS: non-significant, *: p ≤ 0.05, **: p ≤ 0.01.

## Data Availability

All original data will be made available upon request.

## Abbreviations

CRT: Chemoradiotherapy
OARs: Organs at risk
RT: Radiotherapy
VMAT: Volumetric-modulated arc radiotherapy
IMRT: Intensity modulated radiotherapy
HI: Homogeneity index
CI: Conformity index
NPC: Nasopharyngeal carcinomas
MCD: Median cochlear dose
TPS: Treatment Planning System
CCW: Counterclockwise arcs
CW: Clockwise arcs
RAM: Rapid arc model
LKB: Lyman–Kutcher–Burman model
MU: Monitor units
NTCP: Normal tissue complication probability
SNHL: Sensorineural hearing loss
