# Supplementary figures and images for: Cochlea sparing optimized radiotherapy for nasopharyngeal carcinoma
Source: Radiat Oncol. 2021 Apr 1;16:64. doi: 10.1186/s13014-021-01796-4 (PMC8017833; doi:10.1186/s13014-021-01796-4)

**Figure S1:**


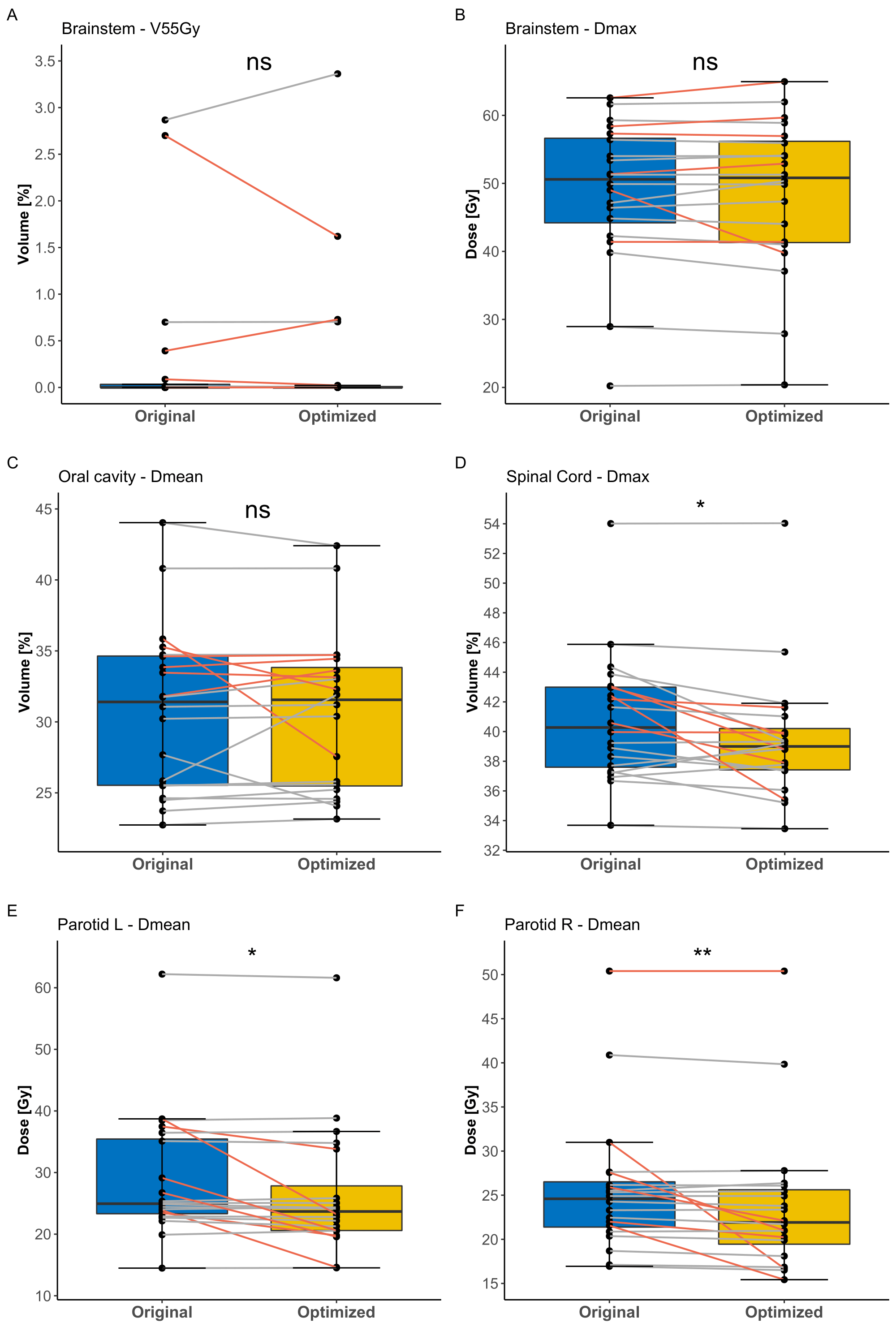


**Figure S2**:
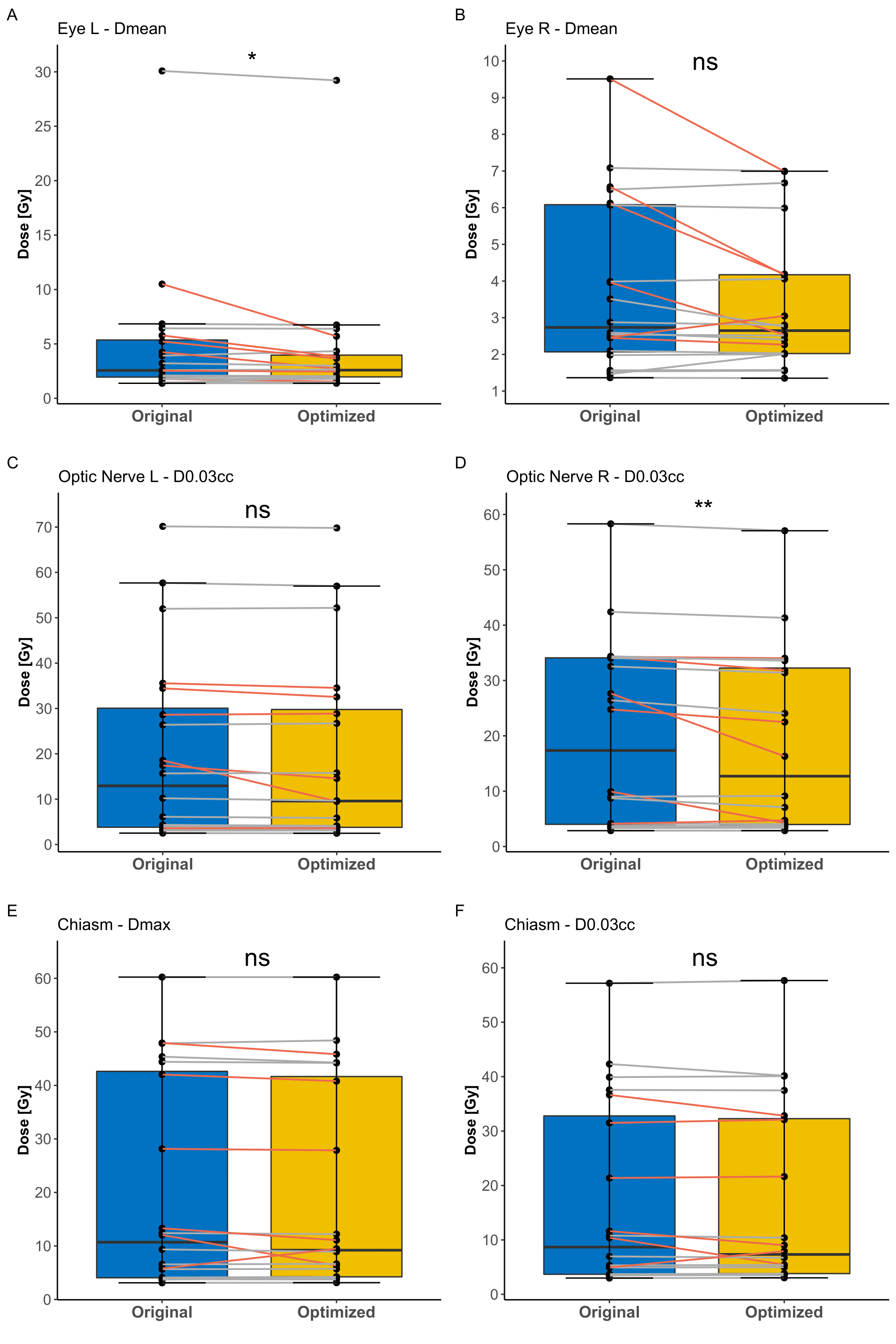

Supplement: Supplementary file 1 — Additional file 1: Figure S1. Boxplots for the dose parameters of brainstem, oral cavity, spinal cord and parotid of the original and optimized plans. Red lines indicates the patients that were originally planned with IMRT (n = 6). Stars indicate significance. NS: non-significant, *: p ≤ 0.05, **: p ≤ 0.01. Figure S2: Boxplots for the dose parameters of eye, optic nerve and chiasm of the original and optimized plans.Red lines indicates the patients that were originally planned with IMRT (n = 6). Stars indicate significance. NS: non-significant, *: p ≤ 0.05, **: p ≤ 0.01. [file 13014_2021_1796_MOESM1_ESM.docx]
